# Patient perspectives on colorectal cancer screening and the role of general practice

**DOI:** 10.1186/s12875-019-0997-5

**Published:** 2019-07-29

**Authors:** Lynsey J. Brown, S. Leigh Roeger, Richard L. Reed

**Affiliations:** 1 Torrens University Australia, 88 Wakefield Street, Adelaide, South Australia 5000 Australia; 2 College of Medicine and Public Health, Flinders University of South Australia, GPO Box 2100, Adelaide, South Australia 5001 Australia

**Keywords:** Colorectal cancer, Bowel cancer, Cancer screening, Population screening, Patient-centred care

## Abstract

**Background:**

Colorectal cancer (CRC) is the second most frequent cause of cancer death in Australia. Early detection can reduce incidence and mortality. General practice-based initiatives have been proposed to improve CRC screening rates but to date have had modest impact. As there is limited research into the patient experience of CRC screening decision making, this study explored patient perspectives on CRC screening and the potential role for general practice.

**Methods:**

Ten participants, aged between 50 and 74, from a general practice in South Australia were recruited by practice staff. Semi-structured interviews were conducted. Concurrent data collection and analysis were performed, guided by interpretative phenomenological analysis.

**Results:**

Two key themes were evident: attitudes toward screening and potential roles for general practice. Participants structured the experience of screening in terms of being proactive, ambivalent or avoidant. Roles for general practice centred on tasks as educators, trusted advisors, monitors and screeners. Mixed views on whether general practice involvement was necessary prompted consideration of additional sources of health information and motivation around screening.

**Conclusions:**

Exploration of the patient experience provides insight into how participants make sense of screening and perceived roles for general practice (or other agents) in screening. There is satisfaction with current Government-driven processes but perceived value in general practice playing a complementary part in increasing screening rates. A multifaceted strategy, accounting for attitudes, is required to improve screening and population health outcomes.

## Background

Colorectal Cancer (CRC), also known as bowel or colon cancer, is the third most commonly diagnosed cancer and second most frequent cause of cancer death in Australia [1]. CRC can be detected before symptom development thus national guidelines recommend biennial screening using non-invasive Faecal Occult Blood Tests (FOBT) from the ages of 50–74 years [2]. Based on evidence that FOBT screening can reduce CRC mortality, in 2006 the Australian Government introduced the National Bowel Cancer Screening Program (NBCSP) [1]. The NBCSP targets early detection in average-risk individuals and desires to promote equitable access to screening and facilitate timely, high-quality diagnostic assessment services. Through the NBCSP, free FOBT kits are mailed to eligible Australians; individuals collect small stool samples and mail them to a testing centre. The results are sent to participants and their nominated health professional. Positive results will usually lead to further testing, coordinated by health professionals. However, consistent with international experience in similar programs, the NBCSP screening uptake is low, with only around 40% of individuals completing their kits [3, 4].

### Barriers and enablers to CRC screening

For individuals, consistently identified barriers to CRC screening relate to: lack of time, procrastination, forgetfulness, other priorities, ambivalence, disvalue of testing, low perception of risk, lack of understanding, fear of results, language difficulties, low socioeconomic status, living in rural locations, age (below 60, over 75), male gender, marital status (single), cultural beliefs and difficulties with the process including disgust at handling samples, concerns about mailing, and anxiety about making mistakes [3, 5–8]. Positive attitudes and prior screening experience have consistently emerged as enabling factors. The latter may refer to FOBT experience, with Australian re-participation rates over 70% [3], with higher re-participation rates believed to be due to familiarity with screening culture and regularly participating in breast/cervical screening [9]. In addition, individuals with higher education, health motivation, and personal or familial cancer experience are more likely to participate. Active screeners cite factors such as the convenience of home testing, desire to prevent illness, maintain health, take advantage of free programs and comply with Government recommendations [10–12]. The most frequently described enabler has been encouragement from others, particularly health professionals [7, 13].

### General practice involvement in CRC screening

Attempts to increase screening have often focused on general practice [14, 15]. Previous research indicates that adherence to screening recommendations is linked with provision of advice from general practice, with an understanding that it is not only the information but the sense of trust, obligation and reciprocity that affects individuals’ confidence in, and understanding of, screening [16, 17]. Subsequently a range of initiatives have been trialled [18]. These have occurred at:

- the policy level, with the recent Primary Health Care Engagement Strategy [19] using partnerships, professional development, resource provision and technological infrastructure support (i.e., National Cancer Screening Register) to encourage the primary care sector to increase participation in the NBCSP;
- the practice level, with initiatives targeting organisational change, general practitioner (GP) attitudes and practice systems (e.g., flags on medical records, letters of endorsement, GP incentives [20–26]); and
- the individual level, with typically positive results for personalised communications, information booklets and phone lines, reminders and interactive educational tools [27–30].

While evidence has demonstrated that general practice interventions can influence screening rates, improvements have been modest, with a recent review [28] reporting an average increase of only 2–3%. It is unclear why these interventions have had only very modest success.

### Patient-centred practices

One proposed answer relates to understanding the patient experience. While many studies exploring barriers and enablers to screening have utilised qualitative methods to understand individuals’ views, intervention studies have traditionally focused on quantitative outcomes such as rates of returned FOBT kits or measures of staff satisfaction [21, 31]. There are few examples in which health service user perspectives on the strategies being trialled (on them), and their acceptability, have been considered.

Current health reforms emphasise the importance of patients driving their own health care. Primary health care is moving toward the ‘patient-centred medical home’ model, an approach which wraps health care provision around patients’ needs and preferences and incorporates shared decision making [29, 32, 33]. In addition, quality improvement is being prioritised, with general practice quality indicators increasingly linked to patient experience [34, 35]. Further, the Australian Government’s National Cancer Screening Register [36] specifically refers to individuals’ ability to control their screening information. Empowering patients is thus an important goal for general practice and screening [37] but at present, there is little evidence of active engagement relating to general practice involvement in CRC screening.

### The current study

The value of engaging general practice in screening seems self-evident but addressing the modest success of interventions requires a pragmatic view on what is required to meet individuals’ needs. In the present patient-centred climate, general practice initiatives should be informed by the perspectives of the service’s users [38]. Therefore, the current study used qualitative methods to address the research question, ‘what do health service users perceive as the role of general practice in CRC screening?’. The aim was to better understand individuals’ experiences of the current NBCSP and the role of general practice in improving participation rates.

## Methods

### Participants and procedure

This study represents the first phase in a program of research examining the role of general practice in CRC screening. Ethics approval was provided by the Torrens University Australia Human Research Ethics Committee. Participants (Table 1), recruited from a general practice in South Australia, included 10 active patients, aged 50–74, eligible for a free FOBT kit. Exclusion criteria included insufficient understanding of English to participate in an interview, poor health, cognitive impairment, and recent personal and/or familial bowel cancer experience. Purposive sampling was used to recruit a maximum variation sample based on age, gender and screening status [39]. Sampling continued until saturation was achieved. In line with previous recommendations [40] the sample size, albeit small, was deemed sufficient to describe the phenomenon in question.

**Table 1:** Participant demographic characteristics

Pseudonym	Age	Gender	Marital status	Education	Employment status	Screening status
Betty	50–54	Female	Married	Completed high school	Employed	Screener
Ed	70–74	Male	Divorced	Some high school	Retired	Screener
Heather	60–64	Female	Married	Completed high school	Employed	Screener
Jenny	60–64	Female	Married	Trade certificate	Employed	Screener
Trevor	65–69	Male	Married	Tertiary qualification	Employed	Screener
John	70–74	Male	Single	Some high school	Employed	Non-screener
Margaret	60–64	Female	Widowed	Completed high school	Employed	Non-screener
Max	70–74	Male	Single	Tertiary qualification	Retired	Non-screener
Megan	55–59	Female	Married	Some high school	Employed	Non-screener
Phil	55–59	Male	Divorced	Trade certificate	Employed	Non-screener

The first author attended the practice on two occasions to complete data collection. At the end of a potentially eligible individual’s appointment, the GP or nurse introduced the research. If the individual was interested, they were introduced to the researcher who provided an information sheet and consent form. The practice was reimbursed AUD$1000 for support with recruitment. Following consent, individuals completed demographic questionnaires and either participated in a semi-structured interview at the practice immediately if convenient (*n* = 9) or scheduled a phone interview (*n* = 1). Interview questions addressed experiences and views on CRC screening and general practice involvement. Participants were reimbursed for their time with an AUD$25 gift voucher.

### Data analysis

Phenomenology focuses on lived experience and the meaning individuals attribute to phenomena; how they make sense of an experience, its structure and essence; and how perceptions impact behaviour [41, 42]. The decision to use this approach was driven by patient-centred approaches and their emphasis on accessing a comprehensive account of what a situation is like for the individual, from their perspective. Interviews were recorded and transcribed in full. Concurrent data collection and analysis were conducted, with emerging themes explored in subsequent interviews. Guided by interpretative phenomenological analysis, themes were drawn from the data, with a focus on description [43–45].

## Results

Themes centred around two key areas: attitudes toward screening and perceived roles for general practice. The following section provides illustrative quotes and further detail; in all cases the names used are pseudonyms. Note that the term ‘general practice’ is inclusive of GPs, practice nurses and administrative staff.

### Attitudes toward screening

Participants were grateful for the Government’s NBCSP, with language around being “lucky” that “they obviously care about everybody” (Megan/55–59/Non-screener). However, CRC was generally an uncomfortable subject to discuss, with participants using vague language to refer to bowels and bowel motions such as “poking around downstairs” (Phil/55–59/Non-screener). Words such as “yucky” and “disgusting” were commonly used, with both verbal and non-verbal cues that discussing bowel issues is taboo.

Whether participants’ first reactions to the kit were acceptance, curiosity or disinterest and whether they perceived the process as simple or complex, depended on their attitudes toward screening. **Proactive** screeners took charge and saw screening as routine and the process as “easy, not a problem” (Jenny/60–64/Screener). These participants were characterised by a tendency to value health, doctors and screening, and a likelihood of participating in other types of screening. They also used more direct language in describing the process.

“Making sure I’m healthy. I’m doing all you know, I do breast screens, I do pap smears, I do the bowel screen and everything” (Betty/50–54/Screener)

“I was somebody who has had health issues for quite a long time. So as I said, I’m open to digging deep to find out what is causing your body to feel whatever way” (Heather/60–64/Screener)

Participants whose experiences were characterised by **ambivalence** acknowledged the usefulness of screening but were not particularly willing to engage. They made comments such as “I was going to get around to it” (Max/70–74/Non-screener). They talked about the process as “awkward” and described actions such as shifting the kit around the house and being uncertain about results: “I don’t want to know but then again I want to know” (Phil/55–59/Non-screener). They described themselves as “casual” or “lazy”. These individuals may have completed one but not subsequent kits, were likely to participate in some other types of screening and seemed to value health.

“I suppose you get the feeling, well you did it once and they didn’t find anything there then. You’re probably not taking as much chance” (Max/70–74/Non-screener)

Those whose lived experience centred on **avoidance** frequently referred to how they believed “the chances of me having that are probably pretty slim” (Megan/55–59/Non-screener), and preferred to be reactive, considering testing only once symptoms were evident. CRC screening was “intrusive”, “uncomfortable”, “intimidating”. These participants had concerns about the process (e.g., “going through the mail they’re going to smell it”; Megan/55–59/Non-screener). There was often an underlying fear of results and their implications. Typically, these individuals had limited knowledge about CRC and reported not participating in other forms of screening.

“They did say I should have bowel screenings often because I could be at risk … but I haven’t. As I said, when I’m left to think about it on my own to do it, I go, ‘oh, nah’ … when you think about it, you could spend your whole life just doing screens … you run the gauntlet anyway, don’t you?” (Margaret/60–64/Non-screener)

“I wouldn’t see a reason why I would need to, unless there were signs or symptoms to say I should. I’m not sure how bowel cancer works, is it a silent type of thing?” (Megan/55–59/Non-screener)

### General practice involvement

#### Roles for general practice

Despite diversity in attitudes, there were consistent roles for general practice identified. The first centred on **education**. Participants described the ability for general practice to provide information, through resources distributed within the practice or by staff. It was acknowledged that such materials need to be brief but adequately detailed to trigger thoughts about the issue.

“Big sign here [points to Doctor’s cupboard] … I guess in waiting rooms they do a lot with the TVs going and talking about cancers and this, that and the other … you can’t imagine people not wanting information that is going to help them” (Heather/60–64/Screener)

GPs’ roles as **trusted advisors** were also recognised. GPs were seen as “your troops in the front” (Ed/70–74/Screener), helping to “keep us well” (Margaret/60–64/Non-screener), knowing individuals’ history and understanding their risks. Some participants believed that if a health professional offered the opportunity to talk face-to-face, explained the process and its benefits and advised them (verbally), they would be more likely to engage in screening.

“If I’d gone to a doctor and they’d said, ‘okay, there is something suspicious going on here’, then I’d definitely, probably wouldn’t hesitate to go ahead with it, because you know, the doctor’s telling me there’s something to worry about” (Megan/55–59/Non-screener)

Multiple participants described **monitoring**, with reminder letters (“you forget things”; John/70–74/Non-screener) or questions during appointments prompting them to complete actions. They suggested these activities be extended to incorporate reminders about CRC screening by using technologies (e.g., practice system alerts, emails or text reminders), or simply asking briefly whether individuals were up-to-date with screening.

“If you’ve got a computer and they can link everything in, it should be linked into that now, so that when you do go, your GP needs to either remind you that one, you either haven’t done it if it hasn’t been sent to you, or even have kits” (Jenny/60–64/Screener)

The ability for **screening** in the practice was mentioned by several participants. This ranged from kit distribution to procuring samples, though the practical restrictions on the latter were mentioned. There was a sense that practice involvement would improve test accuracy: “doing it there and then … there’s more chance of it being effective” (Max/70–74/Non-screener).

“If the patient is able to do it in practice. Then it’s over and done with … You’re kind of forced into a situation. I might be happier, just do it in a practice setting, providing I’m ready to use my bowel of course.” (Megan/55–59/Non-screener)

#### No role for general practice?

When asked whether it was necessary that general practice was involved, many participants said “no”, commenting that the current process “seems to work quite well” (Jenny/60–64/Screener), and that changes to include GPs “wouldn’t make any difference” (Ed/70–74/Screener). It was understood that GPs had priorities other than prevention.

“If they’re going to be involved in bowel cancer screening, then why not be involved in breast cancer screening, and every other type of screening, you know, eye tests, anything, you name it. There’s just a limit” (Margaret/60–64/Non-screener)

“You don’t have that [prevention] conversation generally with the doctor, it’s more or less you’re in for whatever you’re on about and you’re out” (Heather/60–64/Screener)

“It would worry me if the GP send me something … must be something wrong if he’s contacting me to do something, because you only come to see the GP when you’re sick” (Betty/50–54/Screener)

Participants’ perceptions were strongly linked to their attitudes. Ambivalent screeners were more likely to see a role for general practice, acknowledging that they would benefit personally from prompting, reminders and more education. In contrast, avoidant participants saw a benefit for others but not themselves, preferring to circumvent questions by not engaging with the practice (e.g., “I’d like to do it in my own time without being pressured by a phone call”; Megan/55–59/Non-screener). Proactive screeners were also more likely to perceive benefits for others as their personal motivation was internally driven.

“Me personally? No … but I could see the benefits of going through a GP. Some people might feel more comfortable that way. The benefit would be that you can talk to someone about it … I can see a big benefit with different cultures, I can see a lot of people that aren’t really up-to-date with the latest technology where their local GP could explain it to them and break it down” (Phil/55–59/Non-screener)

It was therefore evident that factors beyond general practice influence screening, particularly in relation to sources of information and motivation. Despite the priming effect of sitting in the practice, only two participants mentioned GPs as their main source of health information. Google was consistently cited, while multiple participants also acknowledged pharmacies as useful, especially for those not regularly visiting a general practice.

“Most people go into a chemist quite regularly, for one thing or another, not just for medications. If they had an issue about something and they found a brochure on this or a brochure on that … that would probably hit the public more, I should think, than general practice, and hospitals” (Margaret/60–64/Non-screener)

‘Other people’ were another key source; family, friends and spouses had a significant influence on health behaviours and understanding of issues (e.g., “a friend of mine the other day said he did it, I thought, ‘well, that might encourage me to do it’”; Max/70–74/Non-screener). Further, in addition to medical channels on waiting room televisions, mainstream media emerged as a valuable source, including advertisements, documentaries, talk shows, newspaper articles or radio segments.

Nevertheless, there was consensus that personal stories had the greatest impact on participants’ understanding of health issues, with true stories in the media or from personal connections the biggest influences on intention to screen.

“It wouldn’t matter how many pamphlets they read … if something happened to somebody close to them that would have a far bigger impact” (Ed/70–74/Screener)

“If you saw somebody suddenly having to go to the inconvenience of a bag or something like that … I think those sorts of things probably scare people more when they’ve seen it in reality or get it on a television show” (Max/70–74/Non-screener)

Multiple participants also commented that focusing on general practice activities would only help non-screeners attending primary care. There was a sense that tasks for general practice could be complementary to current processes, or part of a wider strategy appealing to a broader audience.

“You can always have that safety net of the doctor being involved, as well as other places” (Megan/55–59/Non-screener)

## Discussion

Given the low Australian CRC screening rates, in March 2018, the Commonwealth Department of Health, through their Engagement Strategy, released resources encouraging general practices to support the NBCSP [46]. Concurrent reforms in general practice emphasise quality improvement and patient-centred models, empowering patients to be active partners in their care [32]. This qualitative study allowed these voices to be heard. Phenomenology seeks to understand the structure and essence of an experience; how people make sense of it individually and collectively; and the effect of perspectives on behaviour [41, 42]. Previous research has illustrated that individual perceptions, values and attitudes are major predictors of health decisions [29]. It was clear that how participants structured their experience in terms of being proactive, ambivalent or avoidant had a significant impact on screening behaviour and whether they espoused a role for general practice.

Previous literature has highlighted barriers and enablers to CRC screening that were also identified in this study. A unique contribution from this study was the opportunity to connect these factors with attitudes. Procrastination, forgetting, low perceptions of risk, limited understanding of CRC and screening, marital status (i.e., unattached), fear, anxiety and disgust [5–8] were all commonly reported, especially from ambivalent and avoidant screeners. Similarly, participants, particularly proactive screeners, described personal experience, valuing prevention and health, appreciation for free screens and Government support, trust in GPs and high health motivation [10–12, 17] as key enablers. This links to the health belief model which centres on individuals’ perceptions around susceptibility, severity, benefits, barriers and cues to action [47]. In line with this model, proactive screeners had greater understanding of CRC and both its severity and their likely susceptibility. They saw the benefits of screening in ways that overcame any barriers. Ambivalent and avoidant screeners lacked knowledge, being more likely to believe they were not susceptible; demonstrated limited understanding of potential CRC consequences; and disvalued screening. Future initiatives or ‘cues to action’ need to focus on those who are ambivalent and avoidant, tackling barriers and facilitating enablers to encourage screening. Rather than a one-size-fits-all solution, there is a requirement for multifaceted strategies [18, 20, 21] to address different groups’ needs.

While general practice is an obvious context for addressing barriers and enablers, in the current study general practice involvement in screening was not something that participants had previously considered. Participants were not overly enthusiastic; they appreciated the Government-driven process and suggested that the priority in general practice seemed to be acute care rather than prevention. Participants did however identify four key roles that general practice could play in supporting screening: education, advice, monitoring and active screening. In terms of education, a range of tools has been tested with mixed success [18, 21, 23], but maximising use of waiting room facilities and providing resources in this setting were priorities for these participants. As noted previously [28, 30], advice, typically verbal, from GPs or nurses, and letters from the practice were also significant, for both initial endorsement of the FOBT and monitoring/follow-up. General practices as active screening sites were also discussed. While research is exploring this possibility and different types of testing [48], dissemination of kits and offering a collection site for their return may be the only avenues at present. Overall there was potential benefit for general practice involvement, but in contrast to previous research, it was perceived as less important than the need for education and more user-friendly testing processes.

Participants spoke consistently of sources of information and motivation beyond general practice (e.g., Google). Personal stories from other people or in the media had a particularly powerful influence. However, this raises concerns about the accuracy of information. If proactive screening is linked to high health literacy and understanding of CRC and screening, then it is important to consider how to improve adequacy of information and access to trustworthy sources [49], for avoidant and ambivalent screeners in particular. Overall, results from the current study offer insights about lived experiences that suggest that general practice is unlikely to be the complete solution to improving CRC screening rates and that there is a need to consider complementary initiatives that incorporate a range of sources of screening support.

### Limitations and future research

Limitations of this study were predominantly linked to the practice-based nature of the research. There is a risk when practice staff are recruiting that individuals will feel pressured to participate, especially given power dynamics between doctors and patients [39]. It was, however, important that the staff viewed patients’ confidential records to confirm eligibility. Relying on third party recruitment is complex as success is dependent on ‘gatekeeper’ motivation [39]. The environmental influence must also be acknowledged as asking about general practice while in a general practice may be a source of potential bias [39]. However, given that few participants referred to GPs as their first contact for health information it seems this was not an issue. In addition, while generalisability is not the goal for qualitative research [40], it must be acknowledged that these findings may reflect the specific setting or local context (e.g., GP motivations, practice’s current prevention initiatives). Further, the interviewer knew that the Australian Government was increasing responsibility for general practice in CRC screening and subsequently needed to be cautious not to a) direct participants that this is the only choice and b) let this understanding bias interpretations of participants’ preferences.

In terms of future research, this study represents the first phase of a program of research exploring the role of primary health care in promoting CRC screening. Findings from this stage will inform a focus group study and co-design initiative for the development of activities to support CRC screening. Asking similar questions of practice staff and synthesising patient and provider responses with active consultation would be valuable in co-designing practice-based strategies [50, 51]. In addition, though the sample size was typical of phenomenological research [44], future studies may benefit from recruiting greater numbers in order to conduct more detailed comparisons across both attitude-based groups and different practice contexts. Further, negative language describing the FOBT, and the perception that screening could be done within general practice suggests there is a desire for CRC screening to be offered in a different format, potentially one that can be actioned at the point-of-care. Previous literature and participants’ feedback suggest that the current CRC test involves greater effort than other forms of screening [52]. Non-invasive blood tests are being investigated [48] and such initiatives that remove the need to interact with faeces are likely to be of significant benefit. Alternative protocols for test completion (e.g., blood test vs sample, mail out vs return to pharmacy) should be examined in large quantitative trials in future.

### Implications

Recognising the value of health service users’ perspectives is an outcome that could form a core component of future initiatives across various levels. At the individual level, this study offered a unique contribution by providing participants the opportunity to share their experiences. This has helped participants to contribute to informing future CRC screening activities within their local general practice. Active consideration of individuals’ perspectives improves engagement, empowerment and ownership with a service [32]. Further, this research also initiated discussion about CRC thus raising awareness, providing education and encouraging conversations.

At the practice level, results provided insight into individuals’ experiences, offering useful new information to general practice staff. That is, the benefits of resources (i.e., education), endorsement (i.e., advice), electronic or verbal reminders (i.e., monitoring) and active support (i.e., screening) are important for general practices to consider into the future. Not only does including the patient voice align with patient-centred approaches but understanding what users of their service think may help this practice and those with similar populations to decide how to implement the Australian Government’s recommendations in ways that best suit their communities. For example, general practices may review how health promotion materials are shared in their facilities (e.g., waiting room pamphlets, medical channels on waiting room televisions, dissemination of reminders) and whether these methods are directed at the needs of different attitude-based groups.

Individuals need to make well-informed choices regarding screening but with limited knowledge this is an ongoing challenge [53]. A policy level strategy would involve strengthening efforts to disseminate accurate information nationally through key sources such as mainstream media [22]. Such a strategy would allow information to be disseminated broadly across age groups, not focused solely on the cohort eligible for testing. By including youth as targets in campaigns it may help to foster a screening culture within Australia. As it stands, the present results indicate that it is not only understanding the practical process involved in the NBCSP, but the context of CRC and the implications of screening need to be better understood [17, 54]. Personal stories within campaigns are therefore likely to be valuable. This may reduce stigma and raise opportunities for discussion about a topic that is typically taboo. This was demonstrated in a recent UK study recording an increase in public understanding of CRC symptoms following a national media campaign, which saw a 50% increase in patients reporting symptoms to GPs [55]. Additionally, at this policy level it is crucial that the Australian Government engages consumer and practice representatives in developing both future iterations of the NBCSP (and related high-quality information materials) and general practice quality improvement incentives [32, 56].

## Conclusion

CRC is the second most frequent cause of cancer death in Australia. Early detection can help to reduce CRC incidence and mortality and general practice-based initiatives have been proposed to improve the poor CRC screening rates currently reported. Exploration of the patient experience provides insight into both attitudes toward screening and perceived roles for general practice (or other agents) in influencing CRC screening. This study allowed the derivation of potential issues to be addressed by future research. It seems there is satisfaction with the Government-driven process and while there was suggestion that general practice involvement may boost screening rates, views from participants indicate that it is not the only solution. As people make sense of screening in different ways, future strategies to drive uptake of FOBT kits must be designed accordingly. Multifaceted strategies are required incorporating policy (e.g., new media campaigns featuring personal stories), practice (e.g., general practice engagement, resources in pharmacies) and individual level initiatives (e.g., increasing individual knowledge) to improve participation in CRC screening and subsequent population health outcomes.

## Data Availability

The datasets analysed during the current study are available from the corresponding author on reasonable request.

## Abbreviations

CRC: Colorectal Cancer
FOBT: Faecal Occult Blood Test
GP: General Practitioner
NBCSP: National Bowel Cancer Screening Program
